# d-Alanyl-d-Alanine Ligase as a Broad-Host-Range Counterselection Marker in Vancomycin-Resistant Lactic Acid Bacteria

**DOI:** 10.1128/JB.00607-17

**Published:** 2018-06-11

**Authors:** Shenwei Zhang, Jee-Hwan Oh, Laura M. Alexander, Mustafa Özçam, Jan-Peter van Pijkeren

**Affiliations:** a Department of Food Science, University of Wisconsin—Madison, Madison, Wisconsin, USA; Philipps-Universität Marburg

**Keywords:** Ddl ligase, Lactobacillus, *Lactobacillus reuteri*, counterselection, genome editing, lactic acid bacteria, peptidoglycan, probiotics, vancomycin resistance

## Abstract

The peptidoglycan composition in lactic acid bacteria dictates vancomycin resistance. Vancomycin binds relatively poorly to peptidoglycan ending in d-alanyl-d-lactate and binds with high affinity to peptidoglycan ending in d-alanyl-d-alanine (d-Ala-d-Ala), which results in vancomycin resistance and sensitivity, respectively. The enzyme responsible for generating these peptidoglycan precursors is dipeptide ligase (Ddl). A single amino acid in the Ddl active site, phenylalanine or tyrosine, determines depsipeptide or dipeptide activity, respectively. Here, we established that heterologous expression of dipeptide ligase in vancomycin-resistant lactobacilli increases their sensitivity to vancomycin in a dose-dependent manner and overcomes the effects of the presence of a native d-Ala-d-Ala dipeptidase. We incorporated the dipeptide ligase gene on a suicide vector and demonstrated that it functions as a counterselection marker (CSM) in lactobacilli; vancomycin selection allows only those cells to grow in which the suicide vector has been lost. Subsequently, we developed a liquid-based approach to identify recombinants in only 5 days, which is approximately half the time required by conventional approaches. Phylogenetic analysis revealed that Ddl serves as a marker to predict vancomycin resistance and consequently indicated the broad applicability of the use of Ddl as a counterselection marker in the genus Lactobacillus. Finally, our system represents the first “plug and play” counterselection system in lactic acid bacteria that does not require prior genome editing and/or synthetic medium.

**IMPORTANCE** The genus Lactobacillus contains more than 200 species, many of which are exploited in the food and biotechnology industries and in medicine. Prediction of intrinsic vancomycin resistance has thus far been limited to selected Lactobacillus species. Here, we show that heterologous expression of the enzyme Ddl (dipeptide ligase)—an essential enzyme involved in peptidoglycan synthesis—increases sensitivity to vancomycin in a dose-dependent manner. We exploited this to develop a counterselection marker for use in vancomycin-resistant lactobacilli, thereby expanding the poorly developed genome editing toolbox that is currently available for most strains. Also, we showed that Ddl is a phylogenetic marker that can be used to predict vancomycin resistance in Lactobacillus; 81% of Lactobacillus species are intrinsically resistant to vancomycin, which makes our tool broadly applicable.

## INTRODUCTION

Lactic acid bacteria (LAB) are an extraordinarily genetically diverse group of organisms that play a critical role in the fermentation of foods and beverages (1). Select LAB strains, mostly members of the genus Lactobacillus, have health-promoting properties and are, therefore, sought after for their use as dietary supplements (2, 3). Some LAB members have proven invaluable as industrial cell factories of enzymes and are emerging candidates to produce high-value chemicals such as low-calorie sugars from renewable resources (4–6). Thus, LAB are an integral part of our food, health, agriculture, and biotechnology industries and represent, annually, an multibillion dollar market.

To explore the full potential of applications of LAB, genome editing tools are critically important. However, as with most strains, the efficiency and applicability of genetic tools are strain specific. For example, the van Pijkeren and Britton laboratories developed the technologies single-stranded DNA recombineering and clustered regularly interspaced short palindromic repeat (CRISPR)-Cas-mediated genome editing, respectively, in L. reuteri 6475 (7, 8). So far, we have not been able to amend these tools for application in human isolate L. reuteri DSM 17938 to identify recombinants without the need for selection. These tools are also completely dependent on the availability of an inducible gene expression system, which thus far has been validated in only a few species (9–11). Often, we can fall back on more-conventional genome editing strategies that are based on homologous (i.e., Campbell-type) recombination (12). A site-specific homologous recombination event, which is also referred to as a single-crossover (SCO) event, yields integration of a nonreplicating vector in the target locus to insert or inactivate a gene of interest. And yet major drawbacks of this approach include potential polar effects, instability of the integration vector, the need for antibiotic selection, and the limited number of positive selection markers available. Genome editing by two-step allelic exchange, in which the plasmid backbone with the antibiotic marker is excised, is therefore desirable. However, the second crossover event, also referred to as a double-crossover (DCO) event, is rare; thus, the number of allelic exchange mutants in a population is typically very low.

To identify cells in which a DCO event took place, one can use a counterselection marker (CSM) (13). With this technique, an agent in the bacterial growth medium selects for the loss of a gene, i.e., a CSM, that is present on the suicide vector. In other words, bacteria can grow only in the presence of the selective agent when a DCO has occurred. In the genus Lactobacillus, which contains more than 200 species (14), only two CSMs have been adapted for use in three species. Heterologous expression of a variant of the phenylalanyl-tRNA synthetase α-subunit (ePheS) suppresses growth of L. casei in the presence of the phenylalanine analog *p*-chloro-phenylalanine (*p*-Cl-Phe) (15). ePheS misincorporates *p*-Cl-Phe into cellular proteins during translation, thereby causing cell death. The *upp* gene encoding uracil phophoribosyltransferase (UPRTase) also serves as a CSM in L. casei, as well as in L. acidophilus and L. gasseri (16–18). The UPRTase recognizes the base analog 5-fluorouracil (5-FU) and converts it to 5-fluorouracil monophosphate (5-fluoro UMP), which is metabolized to the thymidylate synthase inhibitor 5-fluoro dUMP. Unfortunately, each of these counterselection systems has disadvantages. The application of ePheS requires the use of minimal or semisynthetic medium to avoid incorporation of the phenylalanine that is present in typical media, whereas the application of the UPRTase requires genome editing in a Δ*upp* background.

In this work, we developed the dipeptide ligase (Ddl) enzyme as a counterselection marker in lactobacilli that are intrinsically resistant to vancomycin. The basis for vancomycin resistance or sensitivity is the makeup of the peptidoglycan. The Ddl dipeptide ligase enzyme generates d-alanyl-d-alanine (d-Ala-d-Ala) termini, for which vancomycin has high-affinity binding (19). Thus, bacteria with d-Ala-d-Ala peptidoglycan termini are sensitive to vancomycin. On the other hand, the Ddl depsipeptide ligase enzyme generates d-alanyl-d-lactate (d-Ala-d-Lac) termini, as in the case of L. plantarum (20), for which vancomycin has low-affinity binding. Thus, bacteria with d-Ala-d-Lac peptidoglycan termini are resistant to vancomycin (21). Pioneering work by the Walsh group demonstrated that the composition of the active site of DdlA determines whether Ddl is a dipeptide or depsipeptide ligase (22). Prior work by the same group demonstrated that modification of the Ddl active site (F261) in the lactic acid bacterium Leuconostoc mesenteroides altered the enzyme activity *in vitro* (23). L. mesenteroides is known to be vancomycin resistant, but the amino acid substitution F261Y in Ddl altered enzyme activity from depsipeptide ligase activity to dipeptide ligase activity. The Ddl dipeptide ligase incorporates d-Ala-d-Ala, which is consistent with a vancomycin-sensitive phenotype. Indeed, modification of the Ddl active site (F258Y) in L. reuteri yielded a vancomycin-sensitive derivative (8). Here, we demonstrated that heterologous expression of Ddl dipeptide ligase functions as a CSM in LAB species on the premise that these bacteria are intrinsically resistant to vancomycin. Phylogenetic analysis of Ddl predicted that 140 of 173 Lactobacillus species are intrinsically resistant to vancomycin, which suggests that our system is broadly applicable. We generated markerless genome modifications in Lactobacillus species for which no CSM was previously described, and we developed a liquid-based approach that, combined with our counterselection system, identifies recombinant genotypes in less than half the time required by conventional approaches.

## RESULTS

### Plasmid expression of DdlF258Y increases vancomycin sensitivity in L. reuteri.

**FIG 1:**
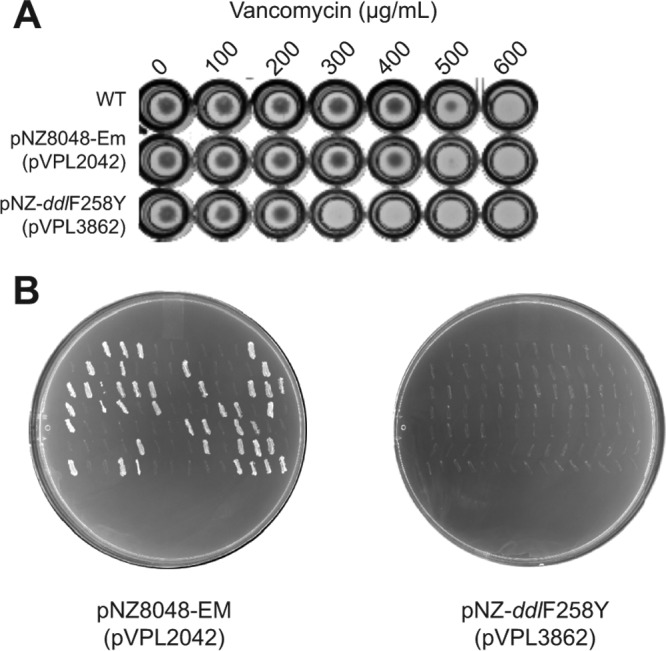
Heterologous expression of DdlF258Y increases vancomycin sensitivity. (A) Plasmid expression of DdlF258Y in L. reuteri 6475 blocked growth in medium containing at least 300 μg/ml vancomycin, whereas growth of wild-type L. reuteri or L. reuteri harboring the control plasmid pVPL2042 was not inhibited in medium containing up to 500 μg/ml vancomycin. Data shown are representative of results from three biological replicates. The differences between replicates in the recorded MICs were no greater than 100 μg/ml. (B) L. reuteri bacteria containing pVPL2042 (left) and pVPL3862 (right) were replica plated from vancomycin to erythromycin plates, with results demonstrating that the presence of DdlF258Y_reuteri_ provides selection in the presence of vancomycin.

First, we showed that L. reuteri harboring pVPL3862, a plasmid that expresses DdlF258Y, did not grow in broth containing 300 μg/ml vancomycin whereas growth of L. reuteri containing the control plasmid (pVPL2042) was not inhibited at up to 500 μg/ml vancomycin (Fig. 1A). To investigate to what extent the combination of vancomycin and DdlF258Y provides negative selection on agar, we replica plated L. reuteri containing pVPL2042 or pVPL3862 from agar plates harboring vancomycin to erythromycin plates. If DdlF258Y prevents growth on plates harboring vancomycin, the only colonies that form on vancomycin plates represent cells that have lost the plasmid, which can be verified by lack of growth on erythromycin. On the basis of three biological replicates, we observed that the pVPL2042 control plasmid was retained at 52% ± 10% following patch plating of 94 colonies from vancomycin to erythromycin plates, which suggests that the control plasmid is unstable in *L. reuteri*. In contrast, none of the L. reuteri colonies that grew on vancomycin plates harbored pVPL3862 (Fig. 1B), which demonstrates that the combination of vancomycin and DdlF258Y provides negative selection on plates. The fact that approximately half the cells had lost the plasmid due to instability—that is, regardless of vancomycin selection conditions—means that the robustness of our assay to determine selectivity in the presence of DdlF258Y and vancomycin was somewhat reduced but that the results remained valid.

### Ddl dipeptide ligase-mediated vancomycin sensitivity can vary depending on the expression host.

Thus far, we had demonstrated the functionality of L. reuteri DdlF258Y in *L. reuteri*. When we introduced *L. reuteri ddl*F258Y—whose expression was under the control of the native L. reuteri promoter—in L. plantarum, we observed poor selection in the presence of vancomycin (data not shown). In addition to poor promoter activity in L. plantarum, differences in codon usage between the two species could affect the translation efficiency. Also, L. plantarum encodes a d-Ala-d-Ala peptidase (Aad), which converts d-Ala-d-Ala substrate into d-Ala to favor formation of d-Ala-d-Lac at the termini of the peptidoglycan side chain (20). Thus, Aad expression may reduce the selective pressure of vancomycin. To circumvent these issues, we fused *ddl*F260Y_plantarum_ to an inducible promoter, which was derived from the sakacin system (24), to yield pSIP-*ddl*F260Y_plantarum_. Indeed, induced expression of DdlF260Y_plantarum_ yielded strong selection in L. plantarum in the presence of vancomycin and reduced the MIC from ≥1,000 μg/ml to 100 μg/ml.

**FIG 2:**
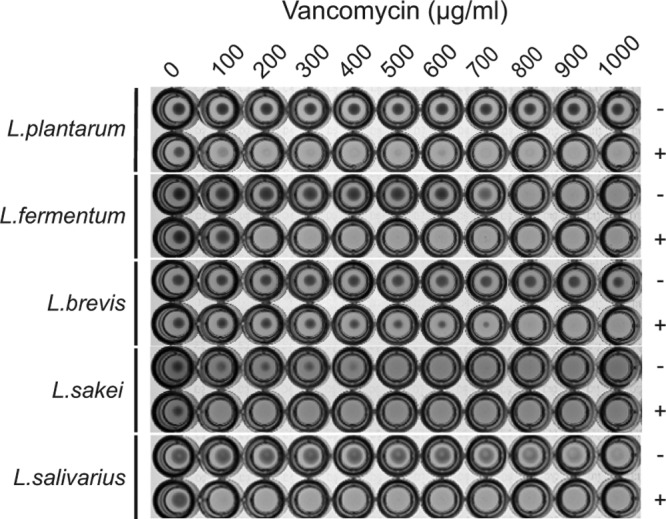
Demonstration of broad-host-range applicability. We tested vancomycin susceptibility of L. plantarum BAA-793, L. fermentum ATCC 14931, L. brevis ATCC 8287, L. sakei ATCC 15521, and L. salivarius CCUG 47825 harboring pSIP-*ddl*F260Y_plantarum_ (pVPL3933) with (+) and without (−) induced expression of DdlF260Y_plantarum_. The vancomycin concentrations used in this assay ranged from 0 to 1,000 μg/ml (left to right). Cultures were also supplemented with 5 μg/ml erythromycin to select for the presence of pSIP-*ddl*F260Y_plantarum_. Data shown are representative of results from three biological replicates, with a maximum MIC variation of ±100 μg/ml.

To assess the activity of L. plantarum DdlF260Y in different hosts, we transformed L. fermentum, L. brevis, L. sakei, and L. salivarius with pSIP-*ddl*F260Y_plantarum_. Our results showed that the levels of reduction of the vancomycin MIC differed depending on the host in which DdlF260Y_plantarum_ was expressed (Fig. 2).

### Sensitivity to vancomycin depends on the Ddl dipeptide transcript level.

**FIG 3:**
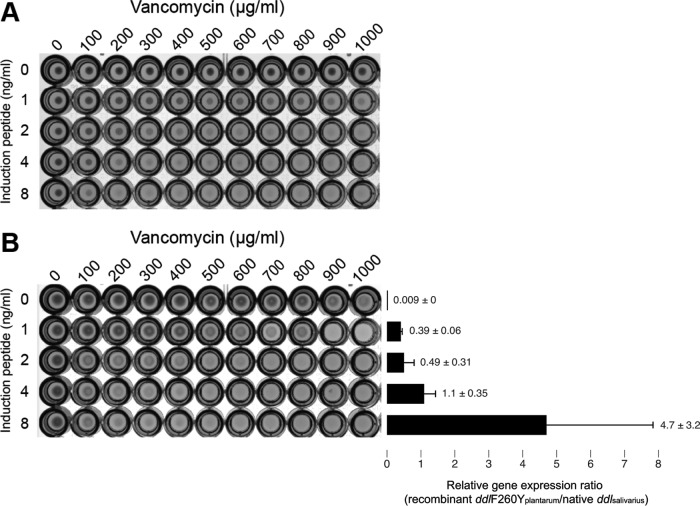
Vancomycin sensitivity depends on the Ddl dipeptide expression level. (A) Assessment of the vancomycin MIC following titration of induction peptide (0, 1, 2, 4, or 8 ng/ml) in L. plantarum BAA-793 harboring pSIP-*ddl*F260Y_plantarum_. (B) (Left panel) Assessment of the vancomycin MIC following titration of induction peptide (0, 1, 2, 4, or 8 ng/ml) in L. salivarius CCUG 47825 harboring pSIP-*ddl*F260Y_plantarum_. Data in panels A and B are representative of results from three biological replicates. The differences between replicates in the recorded MICs were no greater than 100 μg/ml. The pictures of the MIC panels have been modified from the original by removing two rows—corresponding to 0.25 and 0.5 ng/ml induction peptide—which were originally located below the row corresponding to 0 ng/ml induction peptide. (Right panel) We determined for each concentration of the induction peptide the corresponding ratio of *ddl*F260Y_plantarum_ and the native L. salivarius *ddl* transcripts by quantitative real-time PCR. Numbers next to the bars indicate the ratios of the transcript levels of *ddl*F260Y_plantarum_ and L. salivarius *ddl* relative to those of the glyceraldehyde-3-phosphate dehydrogenase (GAPDH) housekeeping gene, which were normalized to the levels determined for the untreated sample (0 ng/ml). Error bars represent standard deviations of results from three biological replicates.

Next, we investigated to what extent expression of recombinant Ddl impacts vancomycin sensitivity. L. plantarum harboring pSIP-*ddl*F260Y_plantarum_ was cultured at different vancomycin concentrations—ranging from 0 to 1,000 μg/ml—in the absence (0 ng/ml) or presence of induction peptide (up to 8 ng/ml) (Fig. 3A). We observed that, compared to the control (0 ng/ml induction peptide), the addition of 0.25 and 0.5 ng/ml did not alter the MIC (data not shown), whereas 1 ng/ml induction peptide reduced growth but not to the point of complete inhibition. Growth was inhibited at 2 and 4 ng/ml induction peptide, resulting in MICs of 900 and 700 μg/ml, respectively, while the MIC was 600 μg/ml at 8 ng/ml. Thus, in L. plantarum, titration of recombinant Ddl expression is correlated to the vancomycin MIC.

To determine the extent to which the transcript ratio of recombinant *ddl* and native *ddl* impacts the vancomycin MIC, we performed quantitative real-time PCR. We could not perform this analysis in L. plantarum harboring pSIP-*ddl*F260Y_plantarum_ because we cannot distinguish between the native and recombinant *ddl* in L. plantarum; these are 99.7% identical. Instead, we performed the analysis on L. salivarius harboring pSIP-*ddl*F260Y_plantarum_. Our data showed that, within the range of 0 to 8 ng/ml induction peptide, the relative transcript level of recombinant *ddl* increased but did not do so in a linear manner. Also, our data showed that when the relative transcript level of recombinant *ddl* was half that seen with the native *ddl*—under conditions of supplementation with 1 or 2 ng/ml induction peptide—the vancomycin MIC was reduced. Supplementation of 8 ng/ml induction peptide increased the recombinant *ddl* transcript level compared to the results seen with 4 ng/ml, and yet, at least in L. salivarius, no difference in the MIC was observed (Fig. 3B).

### The combination of DdlF258Y and vancomycin is a powerful counterselection method in Lactobacillus.

**FIG 4:**
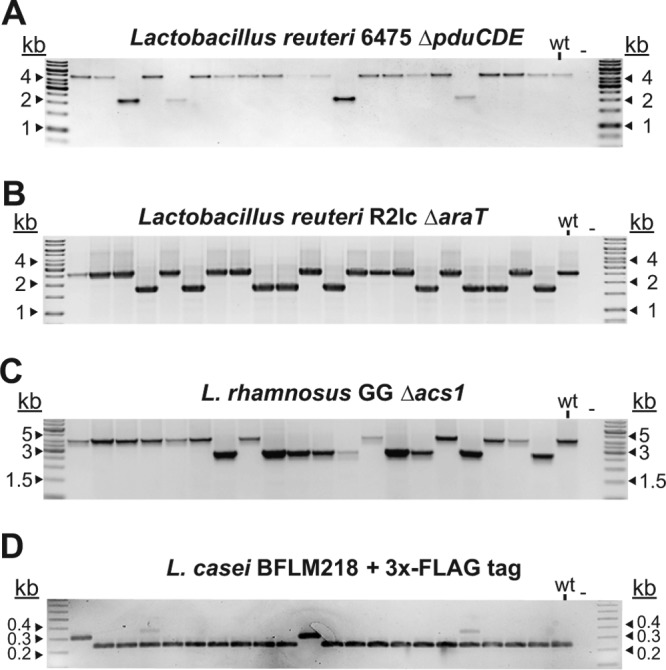
Application of DdlF258Y as a CSM in Lactobacillus. (A) Deletion of *pduCDE* (2.6 kb) in L. reuteri 6475. (B) Deletion of *araT* (870 bp) in L. reuteri R2lc. For both panel A and panel B, vancomycin was used as the counterselection agent to select for cells that had lost the integration vector, i.e., that had undergone a second crossover. We confirmed deletions in L. reuteri 6475 and R2lc in 20% and 45%, respectively, of the screened colonies. We confirmed that all vancomycin-resistant colonies were sensitive to erythromycin. (C) Deletion of acyl-CoA synthetase (1.5 kb) in L. rhamnosus GG was confirmed by colony PCR using oligonucleotides flanking the target region (oVPL192 and oVPL193). (D) Insertion of a 66-bp sequence that encodes the triple FLAG tag at the N-terminal end of PrtP in L. casei BFLM218. Insertions were identified by MAMA-PCR using oVPL505-oVPL506-oVPL507.

Having demonstrated that expression of a dipeptide ligase increased the sensitivity to vancomycin in various species, we explored its potential as a counterselection marker next. To that end, we cloned *L. reuteri ddl*F258Y in pORI19 (25), a LAB suicide vector that we used as a backbone for all Lactobacillus genome editing, to yield pVPL3002. To demonstrate that DdlF258Y can be used as a CSM, we targeted the L. reuteri 6475 *pduCDE* locus encoding diol dehydratase for deletion. We cloned a fusion of the upstream and downstream flanks of the *pduCDE* locus into pVPL3002, which was integrated into the L. reuteri chromosome. We confirmed the SCO genotype using PCR and subsequently cultured the recombinants in the absence of antibiotics, followed by plating on deMan, Rogosa, and Sharpe (MRS) agar with and without vancomycin. We recovered approximately ∼1,000-fold-fewer colonies on vancomycin plates than on the plates lacking antibiotics, which demonstrated selection. The observed selection was driven by the presence of *ddl*F258Y because vancomycin does not suppress growth of wild-type L. reuteri in the absence of *ddl*F258Y. PCR screening confirmed that the colonies on the vancomycin plates had either the wild-type or the recombinant genotype (Fig. 4A). In addition, we used pVPL3002 to generate an in-frame markerless deletion in L. reuteri R2lc. We targeted *araT*, which encodes an aromatic amino acid aminotransferase (26). After vancomycin selection, we performed PCR analyses and confirmed that 9 of 21 colonies had a recombinant genotype (Fig. 4B). Thus, DdlF258Y is applicable as a CSM in two distinct L. reuteri strains.

To extend the application of DdlF258Y as a CSM beyond L. reuteri, we first engineered L. rhamnosus GG (27–30), a probiotic strain for which no CSM is available. We targeted the acyl coenzyme A (acyl-CoA) synthetase gene to generate an in-frame deletion of 1.5 kb. Following identification of a SCO and two passages in MRS to allow a second crossover to occur, we plated cells on vancomycin-containing plates. We confirmed a recombinant genotype by PCR in 9 of 20 colonies (Fig. 4C).

We also demonstrated the applicability of our counterselection system in the human probiotic L. casei BFLM218. We inserted a sequence encoding a triple FLAG tag (3×FLAG) at the N-terminal end of PrtP, also known as lactocepin (31) (Fig. 4D). Subsequent purification of the tagged protein did not yield a biologically active peptidase (Dirk Haller and Gabriele Hörmannsperger, Technische Universität München, personal communication). However, we confirmed the integrity of the DNA sequence by PCR and Sanger sequencing.

### Fast-track genome editing in L. reuteri strains with low transformation efficiencies.

**FIG 5:**
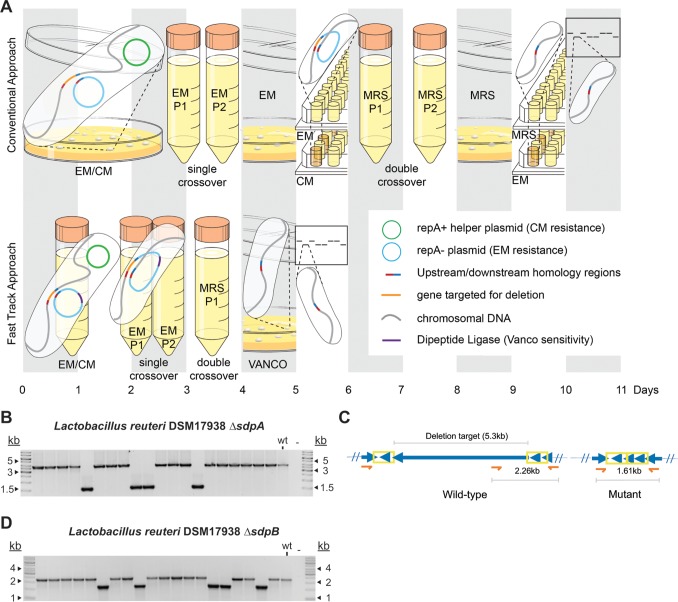
Development of a fast-track genome editing approach. (A) Timeline comparison between the conventional approach and the fast-track liquid approach to generate DCO mutants in L. reuteri 17938. (Top panel) For the conventional approach, the suicide vector (with erythromycin resistance [Em^r^] and lacking *repA* [repA−]) containing homologous target regions was transformed into L. reuteri harboring a helper plasmid (with chloramphenicol resistance [Cm^r^] and *repA* [repA+]) on day 0. After electroporation and recovery, cells were plated on MRS medium containing erythromycin (EM) and chloramphenicol (CM) to select for cells containing both plasmids. At day 2, a single colony was transferred to MRS harboring erythromycin and cultured for a minimum of two passages followed by plating on MRS medium containing erythromycin. At day 5, colonies were tested for Em^r^ and Cm^s^ in broth and were subjected to PCR to confirm SCO integration (not shown). To obtain DCO recombinants, cells were cultured in MRS medium for at least two passages and plated on nonselected plates. At day 9, extensive colony screening in broth was performed to identify Em^s^ cells, i.e., cells in which a DCO event had occurred. Within the pool of Em^s^ cells, PCR was performed to identify the desired genotype (day 11). (Bottom panel) For the fast-track approach, cells harboring the helper plasmid were transformed with the suicide vector encoding the dipeptide ligase (Ddl) enzyme. In bacteria that are intrinsically resistant to vancomycin (VANCO), expression of the dipeptide ligase yields cells that are more sensitive to vancomycin. After recovery, all cells were transferred to MRS broth containing erythromycin and chloramphenicol. After growth had been observed on day 2, cells were washed once in MRS medium and subcultured to MRS medium containing erythromycin for two passages. Cells then underwent one passage in MRS medium followed by plating on MRS medium containing vancomycin (days 3 and 4). The colonies that could grow in the presence of vancomycin had lost the suicide vector encoding Ddl and were subjected to PCR to identify recombinant genotypes. (Illustration by O'Reilly Science Art, LLC.) (B) Deletion of *sdpA* (2.2 kb) in L. reuteri DSM 17938. (C) Oligonucleotide design to identify in-frame deletion in *sdpB* locus. The oligonucleotide combination oVPL2072-oVPL2073-oVPL2694 can identify wild-type or deletion genotypes, yielding an amplicon of 2.2 or 1.6 kb, respectively. Yellow box, homologous region; orange arrow, oligonucleotides. (D) Deletion of *sdpB* (5.3 kb) in L. reuteri DSM 17938.

Efficient genome editing is often strain specific. For example, L. reuteri 6475 and L. reuteri DSM 17938 are human isolates and yet differ in their genome editing potential. We have not been able to adapt single-stranded DNA recombineering in strain DSM 17938. Furthermore, the transformation efficiency of 17938 is 10-fold lower than that of 6475 (unpublished data), which means that we cannot generate plasmid integrants following transformation of a suicide vector. An alternative approach is available that makes use of a helper plasmid (32). A helper plasmid provides RepA in *trans* to support replication of the vector lacking *repA*, which contains DNA homologous to the bacterial target regions. Curing of the helper plasmid, combined with selection for the presence of the vector lacking *repA*, yields single-crossover integrants. A subsequent second homologous recombination event renders either the wild-type or the recombinant genotype (insertion or deletion of the target sequence). However, the complete process to generate markerless mutations in the chromosome is time-consuming, especially without a counterselection system in place (Fig. 5A, top panel). To address this, we developed a fast-track genome editing approach that combines liquid handling with vancomycin-based counterselection (Fig. 5A, bottom panel). Our approach does not require isolation of single-crossover recombinants and/or curing of the helper plasmid as an intermediate step. The rationale is that the DdlF258Y/vancomycin counterselection suppresses the growth of all cells that contain the suicide vector, whether integrated or replicating in the presence of the helper plasmid. Therefore, only a single plating step is required, followed by PCR analysis to identify the recombinant genotype. We developed this fast-track genome editing system for use in L. reuteri DSM 17938 and demonstrated that markerless deletions could be generated in 5 days. First, we generated a 2.2-kb in-frame deletion of the gene encoding sortase-dependent protein A (SdpA) (Fig. 5B). Second, we aimed to delete a 5.3-kb fragment, which corresponds to the gene encoding sortase-dependent protein B (SdpB). To screen for this large deletion, we used a combination of oligonucleotides that were located internally and externally with respect to the target region (Fig. 5C) and confirmed vancomycin selection deletion in 5 of 20 colonies (Fig. 5D).

### Phylogenetic analysis predicts broad applicability of the Ddl dipeptide ligase as a CSM in the genus Lactobacillus.

**FIG 6:**
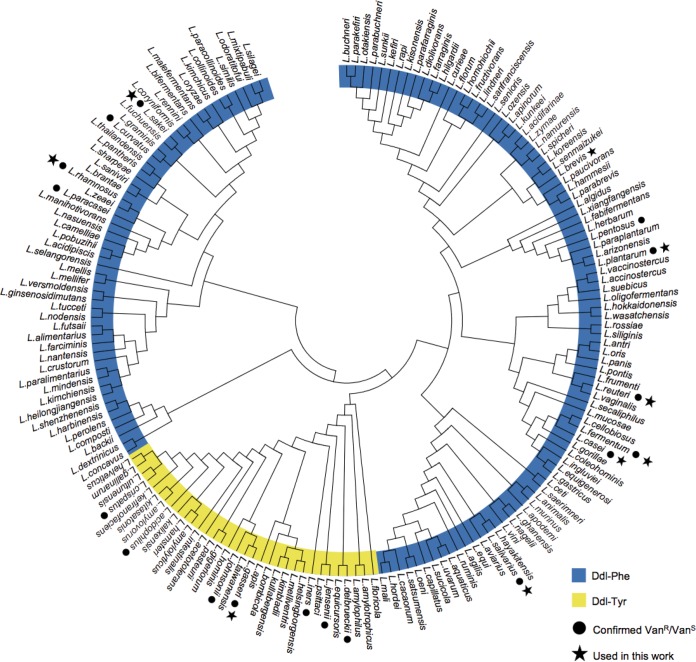
Phylogenetic tree of Lactobacillus DdlA homologs. We constructed a phylogenetic tree based on Ddl sequences derived from 173 Lactobacillus species to predict the vancomycin resistance profile and, thus, the applicability of Ddl as a CSM. Eighty-one percent of Lactobacillus species contain a phenylalanine in the Ddl active site (blue ring), which predicts their intrinsic vancomycin resistance (Van^r^). The remaining species contain a tyrosine in the active site (yellow ring), which predicts vancomycin sensitivity (Van^s^). Our prediction is in accordance with established vancomycin resistance profiles (33–36).

We hypothesized that the Ddl dipeptide ligase is broadly applicable as a CSM in Lactobacillus species on the premise that these species are intrinsically resistant to vancomycin. Indeed, our analysis of Lactobacillus Ddl sequences confirmed that Ddl sequences derived from species that have been experimentally validated to be vancomycin resistant (L. pentosus, L. plantarum, L. reuteri, L. casei, L. fermentum, L. salivarius, L. paracasei, L. rhamnosus, L. curvatus, L. sakei) encode Phe in the active site, while those that are vancomycin sensitive (L. delbrueckii, L. jensenii, L. iners, L. gasseri, L. johnsonii, L. acidophilus, L. crispatus) encode Tyr in the Ddl active site (33–36). To visualize relationships among Lactobacillus Ddl proteins and thus predict vancomycin resistance, we constructed a phylogenetic tree based on Ddl sequences derived from 173 Lactobacillus species (Fig. 6). On the basis of our analysis, only 33 of 173 (19%) Lactobacillus species encoded a tyrosine in the Ddl active site, which suggests dipeptide activity to yield vancomycin sensitivity. Most Lactobacillus species that we investigated (140 of 173; 81%) are predicted to be intrinsically resistant to vancomycin based on the presence of phenylalanine in the Ddl active site. Our prediction is in line with established vancomycin resistance profiles for select species. Thus, Ddl is a marker to predict vancomycin resistance in Lactobacillus, and we expect that Ddl dipeptide ligase can be exploited as a CSM in most Lactobacillus species.

### Potential to use dipeptide ligase as a CSM beyond Lactobacillus.

**FIG 7:**
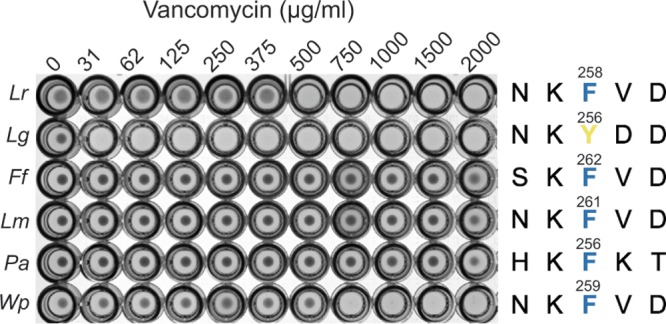
Demonstration of broad-host-range applicability. (A) Vancomycin susceptibility testing of other LAB. The strains used in this assay included L. reuteri 6475 (*Lr*), L. gasseri ATCC 33323 (*Lg*), Fructobacillus fructosus DSM 20349 (*Fr*), Leuconostoc mesenteroides DSM 20346 (*Lm*), Pediococcus acidilactici DSM 20294 (*Pa*), and Weissella paramesenteroides DSM20288 (*Wp*). On the basis of our bioinformatic analysis, the species in the genera Fructobacillus, Leuconostoc, Pediococcus, and Weissella contain a phenylalanine (F) in the Ddl active site (the numbers indicate the positions of the amino acids in Ddl), which matches the observed intrinsic vancomycin resistance profile. Data shown are representative of results from three biological replicates; no differences in MIC were observed between replicates.

We investigated the potential to use the dipeptide ligase as a negative marker in genera beyond Lactobacillus. We expanded our analysis of the Ddl active site to core members of the LAB group, which belong to the order Lactobacillales. These include the genera Fructobacillus, Leuconostoc, Pediococcus, Weissella, Oenococcus, Streptococcus, Aerococcus, Carnobacterium, Enterococcus, Vagococcus, Sporolactobacillus, Tetragenococcus, and Lactococcus. The vancomycin resistance profiles of some LAB, e.g., Leuconostoc spp., Pediococcus spp., and Lactococcus spp., are already known (37, 38), so we included these in our analyses as controls. On the basis of our analyses, only members of the genera Fructobacillus, Leuconostoc, Pediococcus, Weissella, and Oenococcus were found to contain a phenylalanine in the Ddl active site; these are predicted to be intrinsically resistant to vancomycin, which we confirmed for all but Oenococcus (Fig. 7). The genetic toolbox for these genera is limited, but our work has provided a CSM that can be applied once efficient electroporation protocols and plasmid integration protocols have been developed for use in these genera.

## DISCUSSION

Here, we have provided insights into the role of the Ddl dipeptide ligase in vancomycin resistance in lactic acid bacteria and established Ddl as a marker to predict vancomycin resistance in Lactobacillus. In addition, we developed the Ddl dipeptide as a CSM in LAB strains that encode intrinsic vancomycin resistance.

After we established a proof of concept that heterologous expression of dipeptide ligase in L. reuteri increases the sensitivity to vancomycin, we set out to increase our understanding of the dynamics of the correspondence between expression of the Ddl dipeptide ligase and vancomycin sensitivity. In both L. plantarum and L. salivarius, the sensitivity to vancomycin was gradually increased when cells were exposed to increasing levels of induction peptide. In L. salivarius, we confirmed by quantitative real-time PCR that the increase in induction peptide is directly linked to an increase in the transcript level of recombinant Ddl. At least up to the addition of 4 ng/ml induction peptide, which corresponded to equal transcript levels of recombinant *ddl* and native *ddl*, the MIC of vancomycin was gradually reduced. At 8 ng/ml, we observed on average 4.7-fold more transcripts of recombinant *ddl* than of native *ddl*, and yet the MIC was unchanged from that seen at 4 ng/ml. Although the assumption is easily made that increased transcript levels automatically mean more protein production, several studies have demonstrated that—especially at high transcript levels—there can be a disconnection between mRNA and protein levels, at least in Escherichia coli and yeast species (39, 40). Also, the gene encoding DdlF260Y_plantarum_ was not codon optimized for expression in L. salivarius. This could mean that the cell is limited in its ability to translate the recombinant *ddl* transcript due to the lack of select tRNAs. If we were to assume—for the sake of argument—that the increase in the concentration of induction peptide from 4 to 8 ng/ml would yield higher recombinant Ddl levels in the cell, it may be that the protein-folding machinery became saturated, thereby imposing a bottleneck with respect to the amount of biologically active protein produced. The underlying mechanism remains speculative; however, our results do suggest that there is a correlation between Ddl levels and vancomycin resistance. This does raise an interesting question. Can strain-to-strain differences in the MIC of vancomycin be attributed to differential expression levels of the native depsipeptide ligase?

We used a phylogenetic approach to gain more insight into the distribution of putative depsipeptide ligases in the genus Lactobacillus and thus into vancomycin resistance. The accumulation of experimental evidence (i.e., antibiotic tests) over the last 3 decades, combined with the more recent elegant biochemical characterization studies of the peptidoglycan composition in L. plantarum described by members of the Hols group (20, 41), have collectively painted a picture based on the idea that most lactobacilli, with exception of some species, including L. acidophilus and L. delbrueckii, are intrinsically resistant to vancomycin (Fig. 6). However, the vancomycin resistance profile has been described for relatively few species, as the species that were historically isolated and tested were similar. Consequently, the current vancomycin resistance profile covers less than 10% of the Lactobacillus species known to date. To provide an overview of the predicted vancomycin resistance profile at the genus level and, thus, to provide insight into how broadly applicable our counterselection system would be, we performed phylogenetic analysis on the Ddl protein sequence derived from 173 Lactobacillus species. In all Ddl sequences analyzed, the active site was conserved and contained either tyrosine or phenylalanine. Our phylogenetic tree of Ddl of Lactobacillus spp. is unrooted, so we were not able to infer a common ancestor, but we could deduce the relationships between strains on the basis of Ddl sequences, with the differences clearly being driven by the vancomycin resistance profile. Indeed, there was clear clustering of the species that code for tyrosine (Y) in the Ddl active site, which are vancomycin-sensitive species. Interestingly, all vancomycin-sensitive species clustered in the same clade based on the extensive rooted phylogenetic analysis that was performed by Sun et al. (14) and belonged to a deep-branching phylogenetic subgroup within the genus Lactobacillus (42). We propose that Ddl can be a phylogenetic marker to determine whether a species is a member of this deep-branching subgroup.

We have developed a liquid-based approach requiring only limited hands-on time to identify recombinants. Only a single plating is needed, and the time to generate markerless genome modifications has been trimmed to 5 days. That is substantially less time than is needed to generate a markerless mutation using the conventional approach. For example, in L. salivarius, the application of a helper plasmid combined with pORI19 using the conventional approach, without the use of a CSM, generated a markerless deletion in ∼12 days (43). Obviously, the time to generate markerless mutations partially depends on the instability of the helper plasmid and the growth rate of the organism under investigation, but these limitations apply to both the conventional approach and the fast-track approach with the CSM.

Lastly, we want to provide the reader with a brief outline explaining how to optimize this system for use with their vancomycin-resistant lactic acid bacterium of choice, should optimization be necessary. Although we demonstrated the efficacy of the use of L. reuteri DdlF258Y as a CSM in different species, the levels of selective pressure of L. reuteri DdlF258Y differ between species. To address this, the following steps can be taken. First, identify a relative strong promoter. As also evident from our results, increased levels of the Ddl dipeptide ligase provide a more stringent selection. Promoters may not yet have been defined for your strain of interest, but a good starting point is to explore the promoter driving expression of the gene encoding the TU translation elongation factor (P_tuf_). These promoters have been shown to yield strong constitutive expression in L. plantarum and L. buchneri (44), as well as in L. reuteri (Robert Britton, personal communication). In our experience, at least in L. reuteri, a short promoter sequence (<200 bp) on the plasmid does not provide enough sequence homology to drive homologous recombination between the plasmid and the chromosome. Second, we suggest the cloning of a derivative of *ddl* encoding a dipeptide ligase that is codon optimized for expression in the organism of interest. The advantage of this—compared to using a derivative of the native *ddl*—is that it reduces the possibility for homologous recombination to occur between *ddl* sequences. Third, and last, the optimal vancomycin concentration for counterselection should be determined, even if the dipeptide ligase has previously been applied in a different strain of the same species. We base this recommendation on the fact that large variations can exist in the MICs of vancomycin between strains; a panel of 24 L. reuteri strains derived from different hosts (human, chicken, pig, mouse, and rat) revealed that the MIC ranged between 300 and 600 μg/ml, whereas the vancomycin MIC for one pig strain was ≥1,000 μg/ml (data not shown).

In conclusion, increasing our knowledge of the extent to which Ddl impacts vancomycin resistance in lactobacilli enabled us to develop Ddl as a broadly applicable counterselection marker in LAB, a group of organisms of industrial and medical importance.

## MATERIALS AND METHODS

### Bacterial strains, plasmids, and media.

All bacterial strains and plasmids used in this study are listed in Table S1 in the supplemental material. Escherichia coli strains were used as a general cloning host and cultured at 37°C in lysogeny broth (LB; Teknova). LAB were grown as static cultures in deMan, Rogosa, and Sharpe (MRS) medium (Difco; BD BioSciences) under hypoxic conditions (5% CO_2_, 2% O_2_) at 37°C (Lactobacillus casei, Lactobacillus fermentum, Lactobacillus plantarum, Lactobacillus reuteri, Lactobacillus rhamnosus, and Lactobacillus salivarius) or under static conditions at 30°C in a conventional aerated incubator (Lactobacillus brevis and L. sakei, Fructobacillus fructosus, Pediococcus acidilactici, Weissella paramesenteroides, and Leuconostoc mesenteroides). Lactobacillus electrocompetent cells were prepared as described previously (7). The protocol for E. coli EC1000 competent cells preparation was adapted from reference 45. When applicable, erythromycin was supplemented at 5 μg/ml for Lactobacillus strains and at 300 μg/ml for E. coli EC1000. Chloramphenicol was supplemented at 5 μg/ml for Lactobacillus.

### Reagents and enzymes.

PCR amplifications for cloning purposes were performed with Phusion Hot Start II DNA polymerase (Thermo Scientific), while we used Choice *Taq* Mastermix (Denville Scientific) for screening purposes. All modification enzymes were purchased from Fermentas. Ligase cycling reactions (LCR) were performed with Ampligase DNA ligase (Epicentre) as described in reference 46. To concentrate DNA after LCR or conventional T4 DNA ligation, we used Pellet Paint coprecipitant (Novagen). All oligonucleotides were purchased from Integrated DNA Technologies and are listed in Table S2.

### Cloning of *ddl*F258Y.

To construct pNZ-*ddl*F258Y_reuteri_, the backbone was amplified from pVPL2042 with oVPL88-oVPL89, yielding a 3-kb fragment. The *ddl*F258Y gene with its native promoter region was amplified from L. reuteri RPRB3003 (8) using oVPL46-oVPL47. Amplicons were purified using a GeneJET PCR purification kit (Thermo Fisher) and quantified by Qubit fluorometric quantitation (Life Technologies). Amplicons were digested with DpnI, and DNA purification and quantification were conducted as described in reference 10. PCR products were mixed at a 1:3 (vector/insertion) molar ratio followed by blunt-end ligation and electroporation in E. coli EC1000. Oligonucleotides oVPL94-oVPL703 flank the insertion and were used to identify the insertion. The integrity of the DNA sequence was confirmed by Sanger sequencing. The resultant construct was named pVPL3862.

To construct pSIP-*ddl*F260Y_plantarum_, we first amplified the L. plantarum BAA-793 *ddl* gene (lp_2345) with oVPL2589-oVPL2590. The *ddl* amplicon was ligated to the pVPL2042 backbone, which was generated with oVPL2591-oVPL2592, to yield construct pVPL3859. We introduced a single base change (oVPL2593-oVPL2594) by PCR to yield the amino acid change F260Y (pVPL3925). Next, we amplified *ddl*F260Y from pVPL3925 and the backbone of pSIP411 (oVPL399-oVPL400). PCR products were purified, quantified, and DpnI treated as described above. DNA was mixed at a 1:3 (vector/insertion) molar ratio, followed by blunt-end ligation and transformation into E. coli EC1000. Insertion (oVPL659-oVPL2641-oVPL660) was confirmed by colony PCR, and the integrity of the DNA was confirmed by Sanger sequencing. The resultant construct (pVPL3933) has *ddlF260Y* under the control of the sakacin-based inducible promoter (24).

### *ddl* expression analysis.

A stationary-phase culture of L. salivarius CCUG 47825 harboring pSIP-*ddl*F260Y_plantarum_ was induced at an optical density at 600 nm (OD_600_) of 0.005 with the following amounts of pSIP411 induction peptide: 0, 1, 2, 4, and 8 ng/ml. At an OD_600_ of 1, cells were harvested by centrifugation and mixed directly with methanol, followed by storage at −80°C. Total RNA was extracted with TRIzol reagent (Life Technologies) (47), followed by 3 h of DNase treatment (RQ1; Promega). Total RNA was quantified using a Qubit 2.0 fluorometer. A 1-μg volume of RNA was subjected to reverse transcription using an iScript cDNA synthesis kit (Bio-Rad). Twofold dilutions (0 to 1,000 pg) of cDNA were used to determine the primer efficiencies as previously described (48). The relative expression levels of two *ddlA* genes (recombinant *ddl*F260Y_plantarum_ and native *ddl*_salivarius_) were determined using a CFX96 real-time system (Bio-Rad). By PCR, we confirmed that the primer pairs were specific for each of the target genes. As a reference gene, we used the glyceraldehyde-3-phosphate dehydrogenase (GAPDH) gene (49). Relative gene expression analysis was performed using CFX Manager software version 2.1 (Bio-Rad) with the Pfaffl method (48). A total of three biological replicates were performed.

### Induction peptide titration.

To test the vancomycin sensitivity at various induction levels in the species L. plantarum BAA-793 and L. salivarius CCUG 47825, cultures were induced with 0, 1, 2, 4, and 8 ng/ml induction peptide at an OD_600_ of ∼0.005 in the presence of vancomycin (0, 100, 200, 300, 400, 500, 600, 700, 800, 900, and 1,000 μg/ml). Cultures were harvested at 12 h. Cells were resuspended, and 200 μl was transferred to a flat-bottom 96-well plate followed by brief centrifugation (2,000 rpm, 2 min). Images were captured using a Bio-Rad ChemiDoc Touch imaging system. A total of three biological replicates were performed.

### Construction of pVPL3002 and its derivatives.

The pORI19 backbone was amplified with oVPL48-oVPL49, and *L. reuteri ddl*F258Y was amplified from L. reuteri RPRB3003 (8) with oVPL46-oVPL47. The PCR products were mixed and fused by blunt-end ligation. The resultant construct was named pVPL3002.

### Cloning of deletion cassettes.

For each target strain, we performed colony PCR to amplify the upstream and downstream flanking regions (500 bp to 1,000 bp), followed by purification. The pVPL3002 backbone was amplified with oVPL187-oVPL188 followed by purification and DpnI treatment. Phosphorylation and LCR were performed as described above. Ligated product was transformed into E. coli EC1000, followed by PCR screening and sequencing to confirm the plasmid integrity. To construct L. rhamnosus GG coenzyme A (CoA) synthase (*acs1*) deletion vector pVPL3010, the 5′- and 3′-flanking region of *acs1* were amplified with oVPL54-oVPL55 and oVPL52-oVPL53, respectively, followed by overlap extension PCR (50) with oVPL169-oVPL170 to generate the *acs1* deletion cassette. The cassette was cloned in the pVPL3002 backbone by Gibson assembly (51) to yield pVPL3010. Table S2 lists all of the oligonucleotides that we used for amplification and screening purposes.

### Cloning of insertion cassettes.

To insert the DNA sequence encoding a FLAG tag into the L. casei BFLM218 genome, we first cloned the target region in pVPL3002. The 2-kb L. casei amplicon (oVPL380-oVPL381) was cloned into the pVPL3002 backbone (oVPL187-oVPL188) by blunt-end ligation, and the resultant construct was named pVPL3125. To insert the DNA sequence encoding the 22-amino-acid 3×FLAG tag, we amplified the backbone of pVPL3125 with oVPL382-oVPL383. By annealing complementary oligonucleotides oVPL384-oVPL385 (98°C for 2 min followed by gradual cooling down to 25°C), we generated a double-stranded DNA fragment that corresponds to the 66-bp sequence encoding 3×FLAG. The backbone of pVPL3125 and the 3×FLAG sequence were ligated by Gibson assembly and transformed into E. coli EC1000. Erythromycin-positive colonies were subjected to PCR screening (oVPL386-oVPL387-oVPL388), and the integrity of the construct was confirmed by Sanger sequencing. The resultant plasmid was named pVPL3137.

### Vancomycin susceptibility test.

Overnight cultures of the following strains were used in this assay: L. reuteri 6475 wild type, L. reuteri 6475 harboring pVPL2042, and L. reuteri 6475 harboring pVPL3862. The vancomycin concentration in this assay ranged from 100 to 600 μg/ml. To select for the presence of pVPL2042 and its derivatives, 5 μg/ml erythromycin was added to the growth medium. Cultures were standardized to an OD_600_ of 4 and subcultured to 96-well plates at 0.1% (vol/vol) inoculum. Cultures were incubated for 24 h.

To test the induced vancomycin sensitivity in the species L. plantarum BAA-793, L. fermentum ATCC 14931, L. brevis ATCC 8287, L. sakei ATCC 15521, L. salivarius CCUG 47825, cells were transformed with pSIP-*ddl*F260Y_plantarum_ (pVPL3933). Cultures were induced with 100 ng/ml induction peptide (MAGNSSNFIHKIKQI FTHR; Peptide 2.0 Inc.) at an OD_600_ of ∼0.005. L. plantarum and L. salivarius were harvested at 12 h. L. fermentum and L. brevis were incubated for 18 to 20 h. L. sakei was harvested after 72 to 96 h.

Fructobacillus fructosus DSM 20349, Leuconostoc mesenteroides DSM 20346, Pediococcus acidilactici DSM 20284, and Weissella paramesenteroides DSM 20288 were grown in MRS medium and incubated at 30°C. Cultures were standardized to an OD_600_ of 4 and inoculated at 0.1% (vol/vol). The tested concentrations of vancomycin were 30, 60, 130, 250, 380, 500, 750, 1,000, 1,500, and 2,000 μg/ml. Experiments were performed three times.

### *ddl*F258Y: selection on agar.

L. reuteri 6475 harboring pVPL2042 and L. reuteri 6475 harboring pVPL3862 were replica plated from MRS plates containing vancomycin (350 μg/ml) onto MRS plates supplemented with 5 μg/ml erythromycin. Plates were incubated for 24 h.

### Homologous recombination genome editing.

To construct L. reuteri 6475 Δ*pduCDE*, 1 μg plasmid DNA (pVPL3478) was transformed into L. reuteri, which was subsequently selected for SCO on MRS plates containing 5 μg/ml erythromycin. SCO was confirmed by PCR with oligonucleotide pairs oVPL1342-oVPL1343 and oVPL1344-oVPL1345 for upstream and downstream integration, respectively. Subsequently, the SCO mutants were cultured for two passages in MRS medium (0.1% [vol/vol]), followed by plating on MRS agar containing 350 μg/ml vancomycin to select for DCO. PCR screening (oVPL1342-oVPL1344) and Sanger sequencing confirmed the deletion of the *pduCDE* gene cluster. The resultant strain was named VPL4073. To generate a 870-bp deletion of *araT* in L. reuteri R2lc, we used the same approach as described for L. reuteri 6475 with 400 μg/ml vancomycin for counterselection. The resultant mutant was named VPL4192.

To construct L. rhamnosus GG Δ*acs1*, 1 μg/ml plasmid DNA (pVPL3010) was transformed into L. rhamnosus GG. SCO was screened by PCR (oVPL192-146-193 for upstream SCO and oVPL192-147-193 for downstream SCO). After two passages in MRS broth, DCO mutants were screened on a MRS plate containing 1,000 μg/ml vancomycin followed by colony PCR (oVPL192-oVPL193) and sequence analysis. The resultant mutant was named VPL4185. For L. casei BFLM218 3×FLAG insertion, 1 μg/ml plasmid DNA (pVPL3137) was transformed into L. casei BFLM218 (10). After confirmation of the SCO (using oVPL506-oVPL504-oVPL507 for upstream SCO and oVPL506-oVPL505-oVPL507 for downstream SCO), we cultured the bacteria for four subsequent passages to allow a second homologous recombination event to occur, followed by plating on MRS agar containing 1,000 μg/ml vancomycin. Colonies were screened by mismatch amplification mutation assay-PCR (MAMA-PCR) (8) (oVPL505-oVPL506-oVPL507), and the insertion was confirmed by Sanger sequencing. The resultant strain was named VPL4013.

### Fast-track genome editing.

To construct L. reuteri DSM 17938 Δ*sdpA* and Δ*sdpB*, 1 μg/ml plasmid DNA (pVPL3612 or pVPL3762) was transformed into L. reuteri DSM 17938 harboring pVE6007. Cells were recovered in 1 ml MRS medium harboring 5 μg/ml chloramphenicol for 3 h and subsequently were transferred into 40 ml MRS medium containing erythromycin and chloramphenicol (5 μg/ml). After 48 h of incubation, cells were washed once with MRS medium, followed by two passages in MRS medium harboring 5 μg/ml erythromycin (0.1% inoculum). To enable a DCO event to occur, cells were washed once with MRS medium and subcultured in MRS medium at 0.1%. Vancomycin (350 μg/ml) was used to select for the DCO mutants followed by PCR screening and sequence analysis to confirm the Δ*sdpA* deletion (oVPL1562-oVPL1563) and the Δ*sdpB* deletion (MAMA-PCR; oVPL2072-oVPL2694-oVPL2073). The resultant mutants were named VPL4171 and VPL4176, respectively.

### Bioinformatic analysis.

We used the L. reuteri JCM112 DdlA protein sequence (LAR_0465) as a query sequence to search the National Center for Biotechnology Information (NCBI) nonredundant protein sequence (nr) and IMG databases to identify homologs in the following genera: Lactobacillus, Weissella, Fructobacillus, Leuconostoc, Pediococcus, Oenococcus, Streptococcus, Aerococcus, Carnobacterium, Enterococcus, Vagococcus, Sporolactobacillus, Tetragenococcus, and Lactococcus (cutoff E value, 1e−6). Partial sequences and sequences that were not assigned a species name were excluded from the data set. For sequence analysis, we used MEGA 7.0 software (52). We employed the embedded program MUSCLE to generate an alignment of Ddl homologs. For all sequences, we manually confirmed the identity of the amino acid (Phe or Tyr) in the Ddl active site. To demonstrate relationships among Lactobacillus Ddl proteins, we constructed a phylogenetic tree based on Ddl sequences using MEGA 7.0 with the Jones-Taylor-Thornton (JTT) model.

### Accession number(s).

The DNA sequence corresponding to Lactobacillus reuteri R2lc *araT* and those corresponding to its flanking genes have been deposited in GenBank (MG822655).

## Supplementary Material

Supplemental material
