# Supplementary material for: d-Alanyl-d-Alanine Ligase as a Broad-Host-Range Counterselection Marker in Vancomycin-Resistant Lactic Acid Bacteria
Source: J Bacteriol. 2018 Jun 11;200(13):e00607-17. doi: 10.1128/JB.00607-17 (PMC5996685; doi:10.1128/JB.00607-17)
Supplement: Supplemental material [file supp_200_13_e00607-17__index.html]

Supplemental material 

# d-Alanyl-d-Alanine Ligase as a Broad-Host-Range Counterselection Marker in Vancomycin-Resistant Lactic Acid Bacteria

## Supplemental material

- Supplemental file 1 -

  Tables S1 (Bacterial strains and plasmids used) and S2 (Oligonucleotides used)

  PDF, 85K
