# Supplementary material for: d-Alanyl-d-Alanine Ligase as a Broad-Host-Range Counterselection Marker in Vancomycin-Resistant Lactic Acid Bacteria
Source: J Bacteriol. 2018 Jun 11;200(13):e00607-17. doi: 10.1128/JB.00607-17 (PMC5996685; doi:10.1128/JB.00607-17)
Supplement: Supplemental material [file JB.00607-17_zjb999094789s1.pdf]

Supplementary Table S1: Bacterial strains and plasmids used in this study

Bacteria and Plasmids	Characteristics†	Source/Reference
**Bacteria**		
E. coli EC1000	Derivative of *E. coli* MC1000 in which *repA* is integrated in chromosome	(1)
L. reuteri ATCC PTA 6475	Human breast milk isolate	BioGaia AB
VPL4073	In-frame deletion of 2 kb *pduCDE* in *L. reuteri* 6475	This work
L. reuteri R2lc	Rat gastrointestinal isolate	(2)
VPL4192	In-frame deletion of 870 bp *araT* in *L. reuteri* R2lc	This work
L. reuteri DSM 17938	Plasmid-cured derivative of *L. reuteri* ATCC 55730	BioGaia AB, (3)
VPL3565	*L. reuteri* DSM 17938 harboring pVE6007	This work
VPL4171	In-frame deletion of 2.2 kb *sdpA* in *L. reuteri* DSM 17938	This work
VPL4176	In-frame deletion of 5.3 kb *sdpB* in *L. reuteri* DSM 17938	This work
L. rhamnosus GG	Human fecal isolate	ATCC
VPL4185	In-frame deletion of 1.5 kb *acs1* in *L. rhamnosus* GG	This work
L. casei BFLM218	Human fecal isolate	(4)
VPL4013	Insertion of 66 bp 3x-FLAG tag *prtP* gene in *L. casei* BFLM218	This work
L. brevis ATCC 8287	Fermented olives isolate	ATCC
VPL3978	*L. brevis* harboring pVPL3933	This work
L. fermentum ATCC 14931	Fermented beets isolate	ATCC
VPL3950	*L. fermentum* harboring pVPL3933	This work
L. plantarum ATCC BAA-793	Human saliva isolate	ATCC
VPL3938	*L. plantarum* harboring pVPL3933	This work
L. salivarius CCUG 47825	Human blood isolate	CCUG
VPL3941	*L. salivarius* harboring pVPL3933	This work
L. sakei ATCC 15521	Starter of sake	ATCC
VPL3952	*L. sakei* harboring pVPL3933	This work
L. gasseri ATCC 33323	Human intestinal isolate	ATCC
Fructobacillus fructosus DSM 20349	Flowers isolate	DSMZ
Leuconostoc mesenteroides DSM 20346	Hansen's dried starter powder	DSMZ
Pediococcus acidilactici DSM 20284	Barley isolate	DSMZ
Weisella paramesenteroides DSM 20288	Meat isolate	DSMZ
Lactococcus lactis subsp. *cremoris* NZ9000	Dairy starter, derivative of MG1363, *pepN::nisRK*	(5)
**Plasmids**		
pORI19	Em^R, repA-	(6)
pSIP411	Em^R, Sakacin-P based expression vector	(7)
pVPL2042	Em^R, pNZ8048 derivative. Cm marker was replaced by Em marker	Van Pijkeren Lab stock
pVPL3002	pORI19 harboring *L. reuteri* derived *ddl*F258Y	This work
pVPL3010	Em^R, derivative of vector pVPL3002 in which the *L. rhamnosus* GG Δ*acs1* deletion cassette was cloned in the MCS site	This work
pVPL3125	Em^R, derivative of vector pVPL3002 in which 2kb 5'- and 3' flanking sequence required for 3x-FLAG tag insertion in *prtP* of *L. casei* BFLM218 was cloned in the MCS site	This work
pVPL3137	Em^R, derivative of pVPL3125 in which protein encodes 3x-FLAG tag was cloned in between the integration cassette of pVPL3125	This work
pVPL3478	Em^R, derivative of vector pVPL3002 in which the *L. reuteri* 6475 *pduCDE* deletion cassette was cloned in the MCS site	This work
pVPL3612	Em^R, derivative of vector pVPL3002 in which the *L. reuteri* 17938 *sdpA* deletion cassette was cloned in the MCS site	This work
pVPL3694	Em^R, derivative of vector pVPL3002 in which the *L. reuteri* R2lc *araT* deletion cassette was cloned in the MCS site	This work
pVPL3762	Em^R, derivative of vector pVPL3002 in which the *L. reuteri* 17938 *sdpB* deletion cassette was cloned in the MCS site	This work
pVPL3859	Em^R, derivative of vector 2042 in which the *L. plantarum* BAA-793 ddl was inserted in the MSC site	This work
pVPL3862	Em^R, derivative of pVPL2042 in which *ddl*F258Y *was cloned in the* backbone of pVPL2042	This work

Plasmid	Characteristics†	Source/Reference
pVPL3925	Em^R, derivative of vector 2042 in which the *L. plantarum* BAA-793 *ddl*F260Ywas inserted in the MSC site	This work
pVPL3933	Em^R, derivative of pSIP411 in which *ddl*F260Y$_{plantarum}$ was cloned under the control of sakacin-based inducible promoter	This work

VPL: Van Pijkeren Lab strain identification number. pVPL: Van Pijkeren Lab plasmid identification number.
†: *pduCDE:* glycerol dehydratase large subunit PduC, glycerol dehydratase medium subunit PduD, glycerol dehydratase small subunit PduE (BAG26151, BAG26150.1, BAG26149.1); *araT*: aromatic amino acid aminotransferase (MG822655); *sdpA*: bifunctional phosphodiesterase (WP_003672341.1); *sdpB*: hypothetical protein and LPXTG-motif cell wall anchor domain protein (WP_013923860.1，AEI56985.1); *acs1*: acyl-CoA synthetase (WP_014570113.1); Em^R: erythromycin-resistant; Cm^R: chloramphenicol resistant. The accession number listed can be found on https://www.ncbi.nlm.nih.gov. *: ATCC: American Type Culture Collection; DSMZ: German Collection of Microorganisms and Cell Cultures; CCUG: Culture Collection University of Gothenburg.

Table S2. Oligonucleotides used in this study

oligo name	sequence (5'-3')†	Target/comment#
oVPL46	/5phos/aggaaatgcatattcgaactggag	Fwd, amplifies *ddl*F258Y of *L. reuteri* 6475 including native promoter region
oVPL47	/5phos/acattgtactcacaacatcctaacc	Rev, amplifies *ddl*F258Y of *L. reuteri* 6475
oVPL48	tatccgctcacaattccacac	Fwd, amplified the backbone of pORI19
oVPL49	acaatttcacacaggaaacagc	Rev, amplified the backbone of pORI19
oVPL52	tcggaattctgattcacttcacacgcatgg	Fwd, 3' u/s flanking region of *L. rhamnosus* GG
oVPL53	cagtcgtaacttacgcttctttgctcatgattcaggactc	Rev, 3' u/s flanking region of *L. rhamnosus* GG
oVPL54	agtcctgaatcatgagcaaagaagcgtaagttacgactg	Fwd, 5' u/s flanking region of *L. rhamnosus* GG
oVPL55	agctctagaaagcgtccttacacgcagatc	Rev, 5' u/s flanking region of *L. rhamnosus* GG
oVPL88	catggtgagtgcctccttataattta	Rev, starting at ATG of NcoI site of pNZ8048
oVPL89	tagttctagagagctcaagctttc	Fwd, starting at 4bp upstream of XbaI site of pNZ8048
oVPL94	agatctagtcttataactatactgacaatag	Fwd, for pVPL3862 screening
oVPL146	tctatcccaactggctcaagg	Internal oligo for d/s SCO screening of pVPL3010 chromosomal integration
oVPL147	actggaaagatccgccaatac	Internal oligo for u/s SCO screening of pVPL3010 chromosomal integration
oVPL169	ggttttcccagtcacgacgttgtaaaacgacggccagtgaattcgagctcagcgtccttacacgcagatccctg	Gibson oligo, combined with oVPL170 for overlap extension PCR to construct *acs1* deletion cassette
oVPL170	tcgaatatgcatttcctacaatttcacacaggaaacagctatgaccatgagattcacttcacacgcatgggc	Gibson oligo, combined with oVPL169 for overlap extension PCR to construct *acs1* deletion cassette
oVPL171	caacaaccataacgcgttcaccagctcagggatctgcgtgtaaggacgctgagctcgaattcactggccgtcg	Amplifies the backbone of pVPL3002 for Gibson assembly
oVPL172	tcagcaccagccctgtcatgaacatgaagcccatgcgtgtgaagtgaatctcatggtcatagctgtttcctgtg	Amplifies the backbone of pVPL3002 for Gibson assembly
oVPL187	taccgagctcgaattcactgg	Rev, amplifies pVPL3002 backbone
oVPL188	atcctctagagtcgacctgc	Fwd, amplifies pVPL3002 backbone
oVPL192	ttatcgtcacgagccaacac	Fwd, combined with oVPL193 for screening pVPL3010 chromosomal integration and screening *acs1* deletion
oVPL193	tcagttggtcaccgaaacag	Rev, combined with oVPL192 for screening pVPL3010 chromosomal integration and screening *acs1* deletion
oVPL194	tgtgctgcaaggcgattaag	Fwd, for pVPL3125 screening
oVPL380	/5phos/ttccttgtaaaagctactttcagtcac	Fwd, amplifies the integration cassette of pVPL3137 from *L. casei* BFLM218
oVPL381	/5phos/atcgttgttgacaatgatcaagcc	Rev, amplifies the integration cassette of pVPL3137 from *L. casei* BFLM218
oVPL382	agtcggataataaactttcgccaatg	Fwd, combined with oVPL383 for amplifying the backbone of pVPL3125, which can be used for Gibson assembly with insertion cassette (oVPL384-oVPL385)
oVPL383	gatgctaaggccaactcgatg	Rev, combined with oVPL193 for screening pVPL3010 chromosomal integration and screening *acs1* deletion
oVPL384	agttaaaacagtcacattggcgaaagtttattatccgactgattataaggatcatgatggcgattataaggatcatgatatcgattataaggatgatgatgataag gatgctaaggccaactcgatggcgaatgtgcaagccgtat	Combined with oVPL385 for self-ligation to form double strand fragment (3x-FLAG tag) which can be cloned in the backbone of pVPL3125

oligo name	Sequence (5'-3') †	Target/comment#
oVPL385	atacggcttgcacattcgccatcgagttggccttagcatccttatcatcatcatccttataatcgatatcatgatccttataatcgccatcatgatccttataatc agtcggataataaactttcgccaatgtgactgttttaact	Combined with oVPL384 for self-ligation to form double strand fragment (3x-FLAG tag) which can be cloned in the backbone of pVPL3125
oVPL386	ttggcgaaagtttattatccgactgattata	Combined with oVPL387-oVPL388 to screen pVPL3137
oVPL387	aactaaaggatcatcactcgcaaatacg	Combined with oVPL386-oVPL388 to screen pVPL3137
oVPL388	atcctaaggacatgttgaggacatcg	Combined with oVPL386-oVPL387 to screen pVPL3137
oVPL399	ggctaaaatctccttgtaatagtattttatag	Fwd, amplifies the backbone of pSIP411
oVPL400	taatctagactcgaggaattcggtac	Rev, amplifies the backbone of pSIP411
oVPL504	agtttattatccgactgattataaggatc	Fwd, combined with oVPL506-oVPL507 for *L. casei* BFLM218 3x-FLAG tag insertion SCO screening
oVPL505	ttagcatccttatcatcatcatcc	Rev, combine with oVPL506-oVPL507 for *L. casei* BFLM218 3x-FLAG tag insertion SCO screening
oVPL506	tagtgttaaccgattttccataggc	Fwd, combined with oVPL507 and oVPL504 to screen *L. casei* BFLM218 3x-FLAG tag insertion screening
oVPL507	tttgaccggttttactggagagcc	Rev, *L. casei* BFLM218 3x-FLAG tag insertion screening
oVPL659	tgccccgttagttgaagaag	Fwd, amplifies the pSIP411
oVPL660	attctgctcccgcccttatg	Rev, amplifies the pSIP411
oVPL703	tgctgctttttggctatcaa	Rev, for pVPL3862 screening
oVPL1335	ggttcttgaaatgtatgatgca	Fwd, 5' u/s flanking region of *L. reuteri* pduCDE
oVPL1336	acaacagccgaacgatttcc	Rev, 5' u/s flanking region of *L. reuteri* pduCDE
oVPL1337	tggtgctagcgaaattggag	Fwd, 3' d/s flanking region of *L. reuteri* pduCDE
oVPL1338	tacgatcttgccattttcaac	Rev, 3' d/s flanking region of *L. reuteri* pduCDE
oVPL1339	agtgtaaaagttgaaaatggcaagatcgtaggttcttgaaatgtatgatgcattgcgtcc	LCR bridging oligo for pORI-*ddl*F258Y::*pduCDE* deletion cassette
oVPL1340	aaacgacggccagtgaattcgagctcggtatggtgctagcgaaattggagataccattgg	LCR bridging oligo for pORI-*ddl*F258Y::*pduCDE* deletion cassette
oVPL1341	agcgcttattggaaatcgttcggctgttgtatcctctagagtcgacctgcaggcatgcaa	LCR bridging oligo for pORI-*ddl*F258Y::*pduCDE* deletion cassette
oVPL1342	agttgatgccggagtacaag	Fwd, u/s SCO screening of pVPL3478 chromosomal integration<br>Also for DCO screening of Δ*pduCDE* in *L. reuteri* 6475
oVPL1343	tggcgtggcttcattgattc	Rev, u/s SCO screening of pVPL3478 chromosomal integration
oVPL1344	acattggttccagactcaccag	Fwd, d/s SCO screening of pVPL3478 chromosomal integration<br>Also for DCO screening of Δ*pduCDE* in *L. reuteri* 6475
oVPL1345	atggctggacgtgaagtagg	Rev, d/s SCO screening of pVPL3478 chromosomal integration
oVPL1562	atatagtagttacgtaactcacccg	Fwd, for screening of Δ*sdpA* in *L. reuteri* DSM 17938
oVPL1563	tggtagatttttgaacgagcattacc	Rev, for screening of Δ*sdpA* in *L. reuteri* DSM 17938
oVPL1754	atccttgccgtatgaatttcgattg	Fwd, 5' u/s flanking region of *L. reuteri* R2lc *araT*
oVPL1755	caagaaatgaaagatgcttatgagc	Rev, 5' u/s flanking region of *L. reuteri* R2lc *araT*
oVPL1756	agtagctggcatgataaattcctc	Fwd, 3' d/s flanking region of *L. reuteri* R2lc *araT*
oVPL1757	tgccacaatctcatttgaggtaatg	Rev, 3' d/s flanking region of *L. reuteri* R2lc *araT*
oVPL1762	aactggacaattccatcgaggtc	Fwd, for screening of Δ*araT* in *L. reuteri* R2lc
oVPL1763	aaatggattcattgggttttgagcc	Rev, for screening of Δ*araT* in *L. reuteri* R2lc
oVPL1758	aacgacggccagtgaattcgagctcggtaatccttgccgtatgaatttcgattgactgtt	LCR bridging oligo for pORI-*ddl*F258Y::*araT* deletion cassette
oVPL1759	tcgttgctcataagcatctttcatttcttgagtagctggcatgataaattcctcctatat	LCR bridging oligo for pORI-*ddl*F258Y::*araT* deletion cassette
oVPL1760	aagagcattacctcaaatgagattgtggcaatcctctagagtcgacctgcaggcatgcaa	LCR bridging oligo for pORI-*ddl*F258Y::*araT* deletion cassette

**oligo name**	**sequence (5'-3')†**	**Target/comment#**
oVPL1555	agtcataaagccaaccaaaaagg	Fwd, 5' u/s flanking region of *L. reuteri* DSM 17938 *sdpA*
oVPL1556	tgcttaatcgtttaaaatgaaatagac	Rev, 5' u/s flanking region of *L. reuteri* DSM 17938 *sdpA*
oVPL1557	tgcataaaaaaactcctctataaatatac	Fwd, 3' u/s flanking region of *L. reuteri* DSM 17938 *sdpA*
oVPL1558	tcataccggcctgatcgg	Rev, 3' u/s flanking region of *L. reuteri* DSM 17938 *sdpA*
oVPL1559	aaacgacggccagtgaattcgagctcggtaagtcataaagccaaccaaaaaggaatttat	LCR bridging oligo for pORI-*ddl*F258Y::*sdpA* deletion cassette
oVPL1560	agtctatttcattttaaacgattaagcatgcataaaaaaactcctctataaatatacaat	LCR bridging oligo for pORI-*ddl*F258Y::*sdpA* deletion cassette
oVPL1561	gtaatatgcgaaccgatcaggccggtatgaatcctctagagtcgacctgcaggcatgcaa	LCR bridging oligo for pORI-*ddl*F258Y::*sdpA* deletion cassette
oVPL2065	aaaaacaactaacaattatctaacagtaacc	Fwd, 5' u/s flanking region of *L. reuteri* DSM 17938 *sdpB*
oVPL2066	gaagactaattctagacaagtg	Rev, 5' u/s flanking region of *L. reuteri* DSM 17938 *sdpB*
oVPL2067	taacgtttggaattccttattcattcc	Fwd, 3' u/s flanking region of *L. reuteri* DSM 17938 *sdpB*
oVPL2068	ttcatgtcatgcgtaaattcaagg	Rev, 3' u/s flanking region of *L. reuteri* DSM 17938 *sdpB*
oVPL2069	aaacgacggccagtgaattcgagctcggtaaaaaacaactaacaattatctaacagtaac	LCR bridging oligo for pORI-*ddl*F258Y::*sdpB* deletion cassette
oVPL2070	taactattcacttgtctagaattagtcttctaacgtttggaattccttattcattcctct	LCR bridging oligo for pORI-*ddl*F258Y::*sdpB* deletion cassette
oVPL2071	cgagttccttgaatttacgcatgacatgaaatcctctagagtcgacctgcaggcatgcaa	LCR bridging oligo for pORI-*ddl*F258Y::*sdpB* deletion cassette
oVPL2072	aacccaccaatcccaagctatg	Fwd, for screening of Δ*sdpB* in *L. reuteri* DSM 17938
oVPL2073	tgcttatcaacaaagtagattgccc	Rev, for screening of Δ*sdpB* in *L. reuteri* DSM 17938
oVPL2589	ctatcagttgccatcgatggcgacg	Fwd, amplifies promoter region of *L. plantarum* BAA-793 Ddl
oVPL2590	ttattccgcgtctaacgcaacgaaa	Rev, amplifies *L. plantarum* BAA-793 Ddl
oVPL2591	aaaataaattataaggaggcactcaccatgctatcagttgccatcgatggcgacgcaggg	Bridging oligo for *L. plantarum* BAA-793 Ddl and pNZ8048 backbone.
oVPL2592	tatgatttcgttgcgttagacgcggaataatagttctagagagctcaagctttctttgaa	Bridging oligo for *L. plantarum* BAA-793 Ddl and pNZ8048 backbone.
oVPL2593	/5phos/tcacaccactcgcatccacgtacttattattatagtcgtagaacg	Rev, modifies F260Y of *ddl* of *L. plantarum* BAA-793
oVPL2594	/5phos/cgttcgaattaccagtcgaattacc	Fwd, amplifies the *ddl* of *L. plantarum* BAA-793
oVPL2694	taatcgttgtagctctcacgcccca	Combined with oVPL2072-oVPL2073 for screening of Δ*sdpB* in *L. reuteri* DSM 17938
oVPL2841	aaacgacgatgcaatcacag	Fwd, amplifies GAPDH gene of *L. salivarius* CCUG 47825. For qPCR.
oVPL2842	cagcaccagttgagtgagga	Rev, amplifies GAPDH gene of *L. salivarius* CCUG 47825. For qPCR.
oVPL2845	cctgctcgtcaaggttcttc	Fwd, amplifies native *ddl* of *L. salivarius* CCUG 47825. For qPCR.
oVPL2846	acggattgcacctaacttgg	Rev, amplifies native *ddl* of *L. salivarius* CCUG 47825. For qPCR.
oVPL2849	tggaatgctcgatcttaggc	Fwd, amplifies *ddl*F260Y of pVPL3933. For qPCR.
oVPL2850	aaacgcgtctagcgacatct	Rev, amplifies *ddl*F260Y of pVPL3933. For qPCR.

oVPL: Van Pijkeren Lab oligonucleotide identification number.
†: /5phos/: indicates oligonucleotides is phosphorylated on 5'-end;
#: LCR: ligase cycling reaction (8). Fwd: forward; Rev: reverse; GAPDH: glyceraldehyde-3-phosphate dehydrogenase (YP_536057.1). *pduCDE:* glycerol dehydratase large subunit PduC, glycerol dehydratase medium subunit PduD, glycerol dehydratase small subunit PduE (BAG26151, BAG26150.1, BAG26149.1); *araT*: aromatic amino acid aminotransferase (MG822655); *sdpA*: bifunctional phosphodiesterase (WP_003672341.1); *sdpB*: hypothetical protein and LPXTG-motif cell wall anchor domain protein (WP_013923860.1，AEI56985.1); *acs1*: acyl-CoA synthetase (WP_014570113.1); The accession number listed can be found on https://www.ncbi.nlm.nih.gov.

References

1. Leenhouts K, Buist G, Bolhuis A, ten Berge A, Kiel J, Mierau I, Dabrowska M, Venema G, Kok J. 1996. A general system for generating unlabelled gene replacements in bacterial chromosomes. Mol Gen Genet 253:217-24.
2. Molin G, Andersson R, Ahrne S, Lonner C, Marklinder I, Johansson ML, Jeppsson B, Bengmark S. 1992. Effect of fermented oatmeal soup on the cholesterol level and the Lactobacillus colonization of rat intestinal mucosa. Antonie Van Leeuwenhoek 61:167-73.
3. Rosander A, Connolly E, Roos S. 2008. Removal of antibiotic resistance gene-carrying plasmids from *Lactobacillus reuteri* ATCC 55730 and characterization of the resulting daughter strain, *L. reuteri* DSM 17938. Appl Environ Microbiol 74:6032-40.
4. von Schillde MA, Hormannsperger G, Weiher M, Alpert CA, Hahne H, Bauerl C, van Huynegem K, Steidler L, Hrncir T, Perez-Martinez G, Kuster B, Haller D. 2012. Lactocepin secreted by Lactobacillus exerts anti-inflammatory effects by selectively degrading proinflammatory chemokines. Cell Host Microbe 11:387-96.
5. Kuipers OP, de Ruyter PGGA, Kleerebezem M, de Vos WM. 1998. Quorum sensing-controlled gene expression in lactic acid bacteria. Journal of Biotechnology 64:15-21.
6. Law J, Buist G, Haandrikman A, Kok J, Venema G, Leenhouts K. 1995. A System to Generate Chromosomal Mutations in Lactococcus-Lactis Which Allows Fast Analysis of Targeted Genes. Journal of Bacteriology 177:7011-7018.
7. Sorvig E, Mathiesen G, Naterstad K, Eijsink VG, Axelsson L. 2005. High-level, inducible gene expression in *Lactobacillus sakei* and *Lactobacillus plantarum* using versatile expression vectors. Microbiology 151:2439-49.
8. de Kok S, Stanton LH, Slaby T, Durot M, Holmes VF, Patel KG, Platt D, Shapland EB, Serber Z, Dean J, Newman JD, Chandran SS. 2014. Rapid and reliable DNA assembly via ligase cycling reaction. ACS Synth Biol 3:97-106.
